# Dichlorido(η^6^-toluene)[tris­(4-methoxy­phen­yl)phosphine]ruthenium(II)

**DOI:** 10.1107/S1600536808002341

**Published:** 2008-01-30

**Authors:** Lei Wang, Xiang-Ge Zhou, Rui-Xiang Li

**Affiliations:** a Key Laboratory of Green Chemistry and Technology, Ministry of Education, College of Chemistry, Sichuan University, Chengdu, Sichuan 610064, People’s Republic of China

## Abstract

In the title compound, [RuCl_2_(C_7_H_8_)(C_21_H_21_O_3_P)], the Ru^II^ atom possesses a pseudo-octa­hedral geometry and the metrical parameters around the metallic core compare well with those of similar three-legged-piano-stool complexes.

## Related literature

For related literature, see: Elsegood & Tocher (1995 ▶); Hafner *et al.* (1997 ▶); Hansen & Nelson (2000 ▶); Therrien *et al.* (2004 ▶); Eapen & Tamborski (1980 ▶); Winkhaus & Singer (1967 ▶); Zhang *et al.* (2006 ▶).
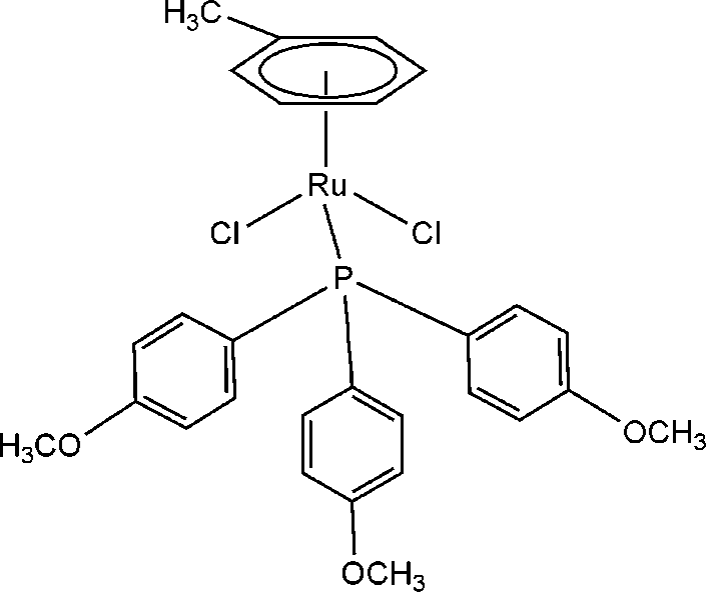


## Experimental

### 

#### Crystal data

[RuCl_2_(C_7_H_8_)(C_21_H_21_O3P)]
*M* *_r_* = 616.45
Orthorhombic, 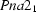

*a* = 22.1789 (2) Å
*b* = 8.0564 (1) Å
*c* = 14.9717 (2) Å
*V* = 2675.17 (5) Å^3^
*Z* = 4
Mo *K*α radiation
μ = 0.87 mm^−1^
*T* = 296 (2) K
0.24 × 0.18 × 0.16 mm

#### Data collection

Bruker SMART CCD area-detector diffractometer
Absorption correction: multi-scan (*SADABS*; Sheldrick, 1996 ▶) *T*_min_ = 0.786, *T*_max_ = 1.000 (expected range = 0.683–0.869)
20209 measured reflections
5872 independent reflections
4769 reflections with *I* > 2σ(*I*)
*R*_int_ = 0.039

#### Refinement

*R*[*F* ^2^ > 2σ(*F* ^2^)] = 0.032
*wR*(*F* ^2^) = 0.074
*S* = 1.01
5872 reflections
320 parameters
1 restraint
H-atom parameters constrained
Δρ_max_ = 0.31 e Å^−3^
Δρ_min_ = −0.38 e Å^−3^
Absolute structure: Flack (1983 ▶), 2694 Friedel pairs
Flack parameter: 0.02 (3)

### 

Data collection: *SMART* (Bruker, 1997 ▶); cell refinement: *SAINT* (Bruker, 1997 ▶); data reduction: *SAINT*; program(s) used to solve structure: *SHELXS97* (Sheldrick, 2008 ▶); program(s) used to refine structure: *SHELXL97* (Sheldrick, 2008 ▶); molecular graphics: *ORTEPIII* (Burnett & Johnson, 1996 ▶) and *ORTEP-3 for Windows* (Farrugia, 1997 ▶); software used to prepare material for publication: *SHELXL97*.

## Supplementary Material

Crystal structure: contains datablocks I, global. DOI: 10.1107/S1600536808002341/dn2314sup1.cif

Structure factors: contains datablocks I. DOI: 10.1107/S1600536808002341/dn2314Isup2.hkl

Additional supplementary materials:  crystallographic information; 3D view; checkCIF report

## Figures and Tables

**Fig. 1.**
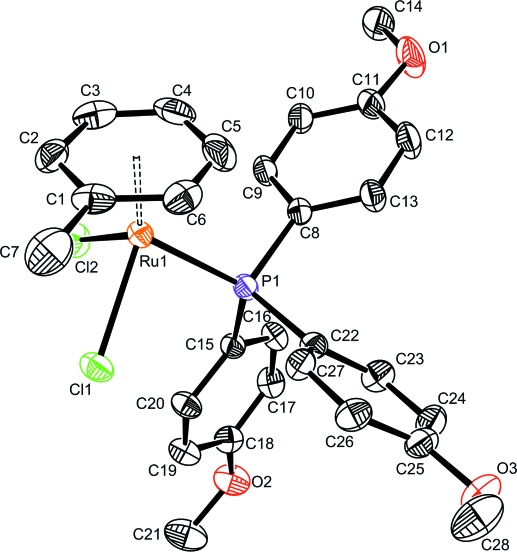
The molecular structure of the title complex with the atom-labelling scheme. Ellipoids are drawn at the 30% probability level. H atoms have been omitted for clarity.

## supplementary crystallographic information

### Comment

Recently, we are interested in the synthesis and catalytic properties of η^6^-arene-ruthenium complexes bearing phosphines (Zhang *et al.*, 2006). These kind of complexes are potential catalysts for many organic reactions, such as hydrogenation of unsaturated organic compound. The title complex was formed in high yield by reacting [RuCl_2_(η^6^-C_6_H_6_)]_2_ with tri(4-methoxyphenyl)phosphine in refluxing toluene. In this reaction, the coordinated benzene in ruthenium was replaced by toluene.

In the title compound, the central Ru atom possesses a pseudo-octahedral geometry and is coordinated by two Cl atoms, one P atom of tri(4-methoxyphenyl)phosphine, and three C=C double bonds of toluene (Fig. 1). The metrical parameters around the metallic core compare well with those of similar three-legged piano-stool [Ru(η6-arene)(PPh3)Cl2] complexes (Elsegood & Tocher, 1995; Hafner *et al.*, 1997; Hansen & Nelson, 2000; Therrien *et al.*, 2004).

### Experimental

Synthetic reaction was performed with standard Schlenk technique under nitrogen atmosphere. Solvents were dried over appropriate drying agents and distilled under nitrogen prior to use. [RuCl_2_(η^6^-C_6_H_6_)]_2_ and tri(4-methoxyphenyl)phosphine were prepared with the reported methods (Eapen & Tamborski, 1980; Winkhaus & Singer, 1967). A mixture of [RuCl_2_(η^6^-C_6_H_6_)]_2_ (0.100 g, 0.20 mmol) and tri(4-methoxyphenyl)phosphine (0.310 g, 0.88 mmol) was refluxed in toluene (50 ml) for 6 h. During refluxing, the solid substances were slowly dissolved and the color of solution changed to crimson. At the end of reaction, the product was obtained as red powder after solvent removal under vacuum. Red crystals of the tittle complex suitable for X-ray structure analysis were obtained by cooling of a dichloromethane-methanol (1:2) solution.

### Refinement

All H atoms were fixed geometrically and treated as riding with C—H = 0.93Å (aromatic) or 0.96 Å (methyl) with *U*_iso_(H) = 1.2*U*_eq_(aromatic) or *U*_iso_(H) = 1.5*U*_eq_(methyl).

### Crystal data

**Table:**

[RuCl_2_(C_7_H_8_)(C_21_H_21_O3P)]	*F*_000_ = 1256
*M**_r_* = 616.45	*D*_x_ = 1.531 Mg m^−^^3^
Orthorhombic, *P**n**a*2_1_	Mo *K*α radiation λ = 0.71073 Å
Hall symbol: P 2c -2n	Cell parameters from 9291 reflections
*a* = 22.1789 (2) Å	θ = 2.3–27.4º
*b* = 8.0564 (1) Å	µ = 0.87 mm^−^^1^
*c* = 14.9717 (2) Å	*T* = 296 (2) K
*V* = 2675.17 (5) Å^3^	Block, red
*Z* = 4	0.24 × 0.18 × 0.16 mm

### Data collection

**Table:**

Bruker SMART CCD area-detector diffractometer	5872 independent reflections
Radiation source: fine-focus sealed tube	4769 reflections with *I* > 2σ(*I*)
Monochromator: graphite	*R*_int_ = 0.039
*T* = 296(2) K	θ_max_ = 27.4º
φ and ω scans	θ_min_ = 1.8º
Absorption correction: multi-scan(SADABS; Sheldrick, 1996)	*h* = −25→28
*T*_min_ = 0.786, *T*_max_ = 1.000	*k* = −10→9
20209 measured reflections	*l* = −19→16

### Refinement

**Table:**

Refinement on *F*^2^	Hydrogen site location: inferred from neighbouring sites
Least-squares matrix: full	H-atom parameters constrained
*R*[*F*^2^ > 2σ(*F*^2^)] = 0.032	*w* = 1/[σ^2^(*F*_o_^2^) + (0.0336*P*)^2^ + 0.3363*P*] where *P* = (*F*_o_^2^ + 2*F*_c_^2^)/3
*wR*(*F*^2^) = 0.074	(Δ/σ)_max_ = 0.006
*S* = 1.01	Δρ_max_ = 0.31 e Å^−^^3^
5872 reflections	Δρ_min_ = −0.38 e Å^−^^3^
320 parameters	Extinction correction: none
1 restraint	Absolute structure: Flack (1983), 2694 Friedel pairs
Primary atom site location: structure-invariant direct methods	Flack parameter: 0.02 (3)
Secondary atom site location: difference Fourier map	

### Special details

**Table:**

Geometry. All e.s.d.'s (except the e.s.d. in the dihedral angle between two l.s. planes) are estimated using the full covariance matrix. The cell e.s.d.'s are taken into account individually in the estimation of e.s.d.'s in distances, angles and torsion angles; correlations between e.s.d.'s in cell parameters are only used when they are defined by crystal symmetry. An approximate (isotropic) treatment of cell e.s.d.'s is used for estimating e.s.d.'s involving l.s. planes.
Refinement. Refinement of *F*^2^ against ALL reflections. The weighted *R*-factor *wR* and goodness of fit *S* are based on *F*^2^, conventional *R*-factors *R* are based on *F*, with *F* set to zero for negative *F*^2^. The threshold expression of *F*^2^ > σ(*F*^2^) is used only for calculating *R*-factors(gt) *etc*. and is not relevant to the choice of reflections for refinement. *R*-factors based on *F*^2^ are statistically about twice as large as those based on *F*, and *R*- factors based on ALL data will be even larger.

### Fractional atomic coordinates and isotropic or equivalent isotropic displacement parameters (Å^2^)

**Table:**

	*x*	*y*	*z*	*U*_iso_*/*U*_eq_	
Ru1	0.028202 (10)	0.41346 (3)	0.250174 (17)	0.03771 (8)	
Cl1	−0.02863 (3)	0.66837 (10)	0.25738 (10)	0.0516 (2)	
Cl2	0.02193 (5)	0.39771 (16)	0.41065 (7)	0.0546 (3)	
P1	0.11610 (3)	0.57488 (10)	0.26850 (6)	0.0369 (2)	
O1	0.33118 (13)	0.1364 (5)	0.3048 (3)	0.0908 (11)	
O2	0.16853 (16)	1.0540 (4)	0.5626 (2)	0.0676 (9)	
O3	0.16755 (16)	1.0654 (4)	−0.0232 (2)	0.0697 (10)	
C1	−0.03640 (19)	0.3238 (5)	0.1463 (3)	0.0566 (11)	
C2	−0.03993 (19)	0.2176 (5)	0.2200 (3)	0.0585 (11)	
H2	−0.0769	0.1954	0.2468	0.070*	
C3	0.01204 (19)	0.1465 (4)	0.2524 (4)	0.0659 (10)	
H3	0.0094	0.0774	0.3020	0.079*	
C4	0.0672 (2)	0.1728 (6)	0.2150 (4)	0.0718 (14)	
H4	0.1011	0.1190	0.2373	0.086*	
C5	0.0722 (2)	0.2804 (7)	0.1433 (4)	0.0750 (15)	
H5	0.1101	0.3023	0.1193	0.090*	
C6	0.0214 (2)	0.3568 (7)	0.1062 (3)	0.0583 (13)	
H6	0.0249	0.4268	0.0571	0.070*	
C7	−0.0898 (3)	0.4094 (7)	0.1111 (4)	0.0903 (17)	
H7A	−0.1088	0.3412	0.0666	0.135*	
H7B	−0.0779	0.5130	0.0848	0.135*	
H7C	−0.1177	0.4299	0.1589	0.135*	
C8	0.18120 (14)	0.4366 (4)	0.2741 (2)	0.0406 (8)	
C9	0.18462 (16)	0.3298 (5)	0.3461 (3)	0.0538 (10)	
H9	0.1529	0.3272	0.3867	0.065*	
C10	0.23391 (16)	0.2258 (5)	0.3599 (3)	0.0554 (11)	
H10	0.2358	0.1569	0.4097	0.067*	
C11	0.27966 (15)	0.2278 (5)	0.2982 (3)	0.0579 (12)	
C12	0.27647 (17)	0.3296 (6)	0.2242 (3)	0.0660 (13)	
H12	0.3071	0.3276	0.1817	0.079*	
C13	0.22733 (16)	0.4352 (5)	0.2131 (3)	0.0503 (9)	
H13	0.2258	0.5054	0.1638	0.060*	
C14	0.3391 (2)	0.0338 (6)	0.3805 (4)	0.0813 (15)	
H14A	0.3379	0.1005	0.4336	0.122*	
H14B	0.3773	−0.0215	0.3767	0.122*	
H14C	0.3074	−0.0473	0.3826	0.122*	
C15	0.12828 (16)	0.7071 (4)	0.3662 (2)	0.0375 (8)	
C16	0.18449 (16)	0.7142 (5)	0.4088 (2)	0.0459 (9)	
H16	0.2146	0.6399	0.3924	0.055*	
C17	0.19565 (17)	0.8287 (5)	0.4743 (3)	0.0501 (9)	
H17	0.2330	0.8305	0.5024	0.060*	
C18	0.15195 (19)	0.9417 (5)	0.4989 (3)	0.0478 (10)	
C19	0.0950 (2)	0.9340 (5)	0.4593 (3)	0.0510 (11)	
H19	0.0648	1.0069	0.4768	0.061*	
C20	0.08419 (17)	0.8170 (5)	0.3939 (3)	0.0455 (9)	
H20	0.0462	0.8119	0.3678	0.055*	
C21	0.1238 (3)	1.1644 (7)	0.5972 (4)	0.0848 (16)	
H21A	0.1107	1.2384	0.5508	0.127*	
H21B	0.1406	1.2275	0.6455	0.127*	
H21C	0.0900	1.1013	0.6185	0.127*	
C22	0.13174 (18)	0.7223 (5)	0.1775 (2)	0.0406 (9)	
C23	0.18352 (19)	0.8211 (5)	0.1782 (3)	0.0532 (10)	
H23	0.2109	0.8122	0.2250	0.064*	
C24	0.19421 (19)	0.9319 (5)	0.1096 (3)	0.0576 (10)	
H24	0.2294	0.9945	0.1095	0.069*	
C25	0.1531 (2)	0.9506 (5)	0.0412 (3)	0.0509 (10)	
C26	0.1007 (2)	0.8580 (5)	0.0417 (3)	0.0539 (11)	
H26	0.0720	0.8721	−0.0031	0.065*	
C27	0.09122 (18)	0.7437 (5)	0.1096 (3)	0.0461 (10)	
H27	0.0563	0.6799	0.1089	0.055*	
C28	0.1281 (3)	1.0834 (7)	−0.0964 (4)	0.0909 (19)	
H28A	0.1224	0.9777	−0.1247	0.136*	
H28B	0.1451	1.1602	−0.1385	0.136*	
H28C	0.0900	1.1248	−0.0759	0.136*	

### Atomic displacement parameters (Å^2^)

**Table:**

	*U*^11^	*U*^22^	*U*^33^	*U*^12^	*U*^13^	*U*^23^
Ru1	0.03768 (12)	0.03808 (13)	0.03737 (14)	0.00240 (10)	0.00388 (15)	−0.00205 (18)
Cl1	0.0416 (3)	0.0481 (4)	0.0652 (6)	0.0107 (3)	−0.0072 (5)	−0.0108 (6)
Cl2	0.0603 (7)	0.0666 (8)	0.0368 (5)	−0.0049 (5)	0.0090 (4)	0.0002 (5)
P1	0.0342 (4)	0.0391 (4)	0.0374 (6)	0.0053 (3)	0.0039 (3)	0.0028 (4)
O1	0.0566 (17)	0.106 (2)	0.110 (3)	0.0447 (17)	0.0301 (17)	0.045 (2)
O2	0.079 (2)	0.068 (2)	0.056 (2)	0.0084 (17)	−0.0140 (16)	−0.0150 (16)
O3	0.085 (2)	0.058 (2)	0.066 (2)	−0.0173 (17)	−0.0039 (17)	0.0198 (17)
C1	0.066 (3)	0.043 (2)	0.060 (2)	0.003 (2)	−0.031 (2)	−0.016 (2)
C2	0.061 (2)	0.049 (2)	0.065 (3)	−0.0174 (19)	0.0051 (19)	−0.006 (2)
C3	0.098 (3)	0.0372 (17)	0.062 (2)	0.0010 (18)	−0.013 (4)	0.003 (3)
C4	0.073 (3)	0.047 (2)	0.095 (4)	0.019 (2)	−0.003 (3)	−0.026 (3)
C5	0.067 (3)	0.079 (4)	0.079 (3)	−0.003 (3)	0.026 (3)	−0.040 (3)
C6	0.086 (4)	0.050 (3)	0.039 (3)	−0.011 (3)	0.002 (2)	−0.006 (2)
C7	0.099 (4)	0.090 (4)	0.081 (4)	−0.020 (3)	−0.013 (3)	0.001 (3)
C8	0.0364 (15)	0.0409 (18)	0.044 (2)	0.0012 (13)	0.0061 (14)	0.0031 (15)
C9	0.044 (2)	0.055 (2)	0.062 (3)	0.0124 (18)	0.0195 (18)	0.016 (2)
C10	0.043 (2)	0.053 (2)	0.070 (3)	0.0092 (18)	0.0145 (18)	0.022 (2)
C11	0.042 (2)	0.055 (3)	0.076 (3)	0.0142 (19)	0.0112 (18)	0.010 (2)
C12	0.046 (2)	0.082 (3)	0.070 (3)	0.020 (2)	0.0230 (19)	0.023 (3)
C13	0.044 (2)	0.061 (2)	0.046 (2)	0.0106 (17)	0.0116 (16)	0.0108 (18)
C14	0.057 (3)	0.070 (3)	0.117 (4)	0.024 (2)	0.012 (3)	0.031 (3)
C15	0.0349 (18)	0.038 (2)	0.0401 (19)	0.0029 (15)	0.0009 (15)	0.0029 (16)
C16	0.0409 (18)	0.054 (2)	0.043 (2)	0.0074 (17)	0.0026 (16)	−0.0038 (18)
C17	0.043 (2)	0.064 (3)	0.043 (2)	0.0007 (19)	−0.0061 (16)	0.001 (2)
C18	0.057 (2)	0.048 (3)	0.038 (2)	0.0019 (19)	−0.0024 (18)	0.0016 (18)
C19	0.054 (2)	0.051 (3)	0.049 (2)	0.014 (2)	−0.0048 (18)	−0.006 (2)
C20	0.0408 (19)	0.048 (2)	0.048 (2)	0.0120 (18)	−0.0079 (17)	−0.0041 (19)
C21	0.111 (4)	0.079 (4)	0.065 (3)	0.027 (4)	−0.015 (3)	−0.023 (3)
C22	0.046 (2)	0.037 (2)	0.039 (2)	0.0029 (17)	0.0057 (16)	0.0002 (16)
C23	0.054 (2)	0.052 (3)	0.054 (2)	−0.003 (2)	−0.0089 (18)	0.0054 (19)
C24	0.053 (2)	0.055 (2)	0.064 (3)	−0.011 (2)	0.004 (2)	0.009 (2)
C25	0.069 (3)	0.038 (2)	0.045 (2)	−0.005 (2)	0.006 (2)	0.0030 (19)
C26	0.061 (3)	0.049 (3)	0.052 (2)	−0.002 (2)	−0.007 (2)	0.006 (2)
C27	0.045 (2)	0.045 (2)	0.049 (2)	−0.0058 (18)	0.0004 (17)	0.0055 (19)
C28	0.132 (5)	0.080 (4)	0.061 (3)	−0.025 (4)	−0.023 (3)	0.026 (3)

### Geometric parameters (Å, °)

**Table:**

Ru1—C5	2.160 (4)	C9—H9	0.9300
Ru1—C3	2.181 (3)	C10—C11	1.372 (5)
Ru1—C4	2.188 (4)	C10—H10	0.9300
Ru1—C6	2.208 (5)	C11—C12	1.382 (5)
Ru1—C2	2.231 (4)	C12—C13	1.392 (5)
Ru1—C1	2.235 (4)	C12—H12	0.9300
Ru1—P1	2.3593 (8)	C13—H13	0.9300
Ru1—Cl2	2.4099 (12)	C14—H14A	0.9600
Ru1—Cl1	2.4121 (8)	C14—H14B	0.9600
P1—C8	1.826 (3)	C14—H14C	0.9600
P1—C15	1.830 (4)	C15—C20	1.382 (5)
P1—C22	1.841 (4)	C15—C16	1.402 (5)
O1—C11	1.363 (4)	C16—C17	1.369 (5)
O1—C14	1.414 (6)	C16—H16	0.9300
O2—C18	1.365 (5)	C17—C18	1.380 (6)
O2—C21	1.429 (6)	C17—H17	0.9300
O3—C25	1.373 (5)	C18—C19	1.396 (6)
O3—C28	1.410 (6)	C19—C20	1.380 (6)
C1—C2	1.399 (6)	C19—H19	0.9300
C1—C6	1.441 (7)	C20—H20	0.9300
C1—C7	1.469 (6)	C21—H21A	0.9600
C2—C3	1.375 (6)	C21—H21B	0.9600
C2—H2	0.9300	C21—H21C	0.9600
C3—C4	1.362 (6)	C22—C27	1.367 (5)
C3—H3	0.9300	C22—C23	1.397 (6)
C4—C5	1.385 (7)	C23—C24	1.381 (6)
C4—H4	0.9300	C23—H23	0.9300
C5—C6	1.399 (7)	C24—C25	1.380 (6)
C5—H5	0.9300	C24—H24	0.9300
C6—H6	0.9300	C25—C26	1.381 (6)
C7—H7A	0.9600	C26—C27	1.388 (6)
C7—H7B	0.9600	C26—H26	0.9300
C7—H7C	0.9600	C27—H27	0.9300
C8—C13	1.372 (4)	C28—H28A	0.9600
C8—C9	1.381 (5)	C28—H28B	0.9600
C9—C10	1.393 (5)	C28—H28C	0.9600
			
C5—Ru1—C3	66.2 (2)	Ru1—C6—H6	129.8
C5—Ru1—C4	37.14 (19)	C1—C7—H7A	109.5
C3—Ru1—C4	36.33 (18)	C1—C7—H7B	109.5
C5—Ru1—C6	37.33 (19)	H7A—C7—H7B	109.5
C3—Ru1—C6	78.4 (2)	C1—C7—H7C	109.5
C4—Ru1—C6	66.9 (2)	H7A—C7—H7C	109.5
C5—Ru1—C2	78.77 (17)	H7B—C7—H7C	109.5
C3—Ru1—C2	36.30 (15)	C13—C8—C9	118.3 (3)
C4—Ru1—C2	65.93 (17)	C13—C8—P1	124.4 (3)
C6—Ru1—C2	67.05 (17)	C9—C8—P1	117.4 (2)
C5—Ru1—C1	67.28 (18)	C8—C9—C10	122.3 (3)
C3—Ru1—C1	65.56 (18)	C8—C9—H9	118.9
C4—Ru1—C1	78.42 (16)	C10—C9—H9	118.9
C6—Ru1—C1	37.83 (17)	C11—C10—C9	118.2 (4)
C2—Ru1—C1	36.51 (16)	C11—C10—H10	120.9
C5—Ru1—P1	89.21 (13)	C9—C10—H10	120.9
C3—Ru1—P1	132.66 (11)	O1—C11—C10	124.4 (4)
C4—Ru1—P1	100.95 (13)	O1—C11—C12	114.9 (4)
C6—Ru1—P1	106.49 (14)	C10—C11—C12	120.6 (3)
C2—Ru1—P1	166.66 (11)	C11—C12—C13	119.9 (3)
C1—Ru1—P1	142.09 (13)	C11—C12—H12	120.1
C5—Ru1—Cl2	137.63 (17)	C13—C12—H12	120.1
C3—Ru1—Cl2	85.62 (18)	C8—C13—C12	120.6 (4)
C4—Ru1—Cl2	102.49 (15)	C8—C13—H13	119.7
C6—Ru1—Cl2	163.37 (14)	C12—C13—H13	119.7
C2—Ru1—Cl2	97.20 (11)	O1—C14—H14A	109.5
C1—Ru1—Cl2	129.78 (13)	O1—C14—H14B	109.5
P1—Ru1—Cl2	87.75 (4)	H14A—C14—H14B	109.5
C5—Ru1—Cl1	133.79 (17)	O1—C14—H14C	109.5
C3—Ru1—Cl1	138.86 (12)	H14A—C14—H14C	109.5
C4—Ru1—Cl1	166.42 (13)	H14B—C14—H14C	109.5
C6—Ru1—Cl1	100.61 (15)	C20—C15—C16	117.8 (3)
C2—Ru1—Cl1	104.91 (11)	C20—C15—P1	120.5 (3)
C1—Ru1—Cl1	88.35 (11)	C16—C15—P1	121.3 (3)
P1—Ru1—Cl1	87.55 (3)	C17—C16—C15	120.9 (3)
Cl2—Ru1—Cl1	88.29 (5)	C17—C16—H16	119.5
C8—P1—C15	101.64 (15)	C15—C16—H16	119.5
C8—P1—C22	106.18 (16)	C16—C17—C18	120.6 (4)
C15—P1—C22	100.85 (16)	C16—C17—H17	119.7
C8—P1—Ru1	108.81 (11)	C18—C17—H17	119.7
C15—P1—Ru1	122.41 (12)	O2—C18—C17	115.8 (4)
C22—P1—Ru1	115.17 (13)	O2—C18—C19	124.8 (4)
C11—O1—C14	118.5 (3)	C17—C18—C19	119.5 (4)
C18—O2—C21	118.6 (4)	C20—C19—C18	119.3 (4)
C25—O3—C28	118.0 (4)	C20—C19—H19	120.3
C2—C1—C6	119.4 (4)	C18—C19—H19	120.3
C2—C1—C7	121.7 (4)	C19—C20—C15	121.8 (4)
C6—C1—C7	118.8 (5)	C19—C20—H20	119.1
C2—C1—Ru1	71.6 (2)	C15—C20—H20	119.1
C6—C1—Ru1	70.1 (2)	O2—C21—H21A	109.5
C7—C1—Ru1	128.0 (3)	O2—C21—H21B	109.5
C3—C2—C1	119.1 (4)	H21A—C21—H21B	109.5
C3—C2—Ru1	69.9 (2)	O2—C21—H21C	109.5
C1—C2—Ru1	71.9 (2)	H21A—C21—H21C	109.5
C3—C2—H2	120.5	H21B—C21—H21C	109.5
C1—C2—H2	120.5	C27—C22—C23	118.3 (4)
Ru1—C2—H2	130.2	C27—C22—P1	120.5 (3)
C4—C3—C2	122.9 (5)	C23—C22—P1	121.1 (3)
C4—C3—Ru1	72.1 (2)	C24—C23—C22	120.2 (4)
C2—C3—Ru1	73.8 (2)	C24—C23—H23	119.9
C4—C3—H3	118.5	C22—C23—H23	119.9
C2—C3—H3	118.5	C25—C24—C23	120.6 (4)
Ru1—C3—H3	127.8	C25—C24—H24	119.7
C3—C4—C5	119.2 (4)	C23—C24—H24	119.7
C3—C4—Ru1	71.5 (2)	O3—C25—C24	116.1 (4)
C5—C4—Ru1	70.3 (3)	O3—C25—C26	124.3 (4)
C3—C4—H4	120.4	C24—C25—C26	119.6 (4)
C5—C4—H4	120.4	C25—C26—C27	119.3 (4)
Ru1—C4—H4	130.2	C25—C26—H26	120.3
C4—C5—C6	121.2 (4)	C27—C26—H26	120.3
C4—C5—Ru1	72.5 (3)	C22—C27—C26	121.9 (4)
C6—C5—Ru1	73.2 (3)	C22—C27—H27	119.0
C4—C5—H5	119.4	C26—C27—H27	119.0
C6—C5—H5	119.4	O3—C28—H28A	109.5
Ru1—C5—H5	126.9	O3—C28—H28B	109.5
C5—C6—C1	118.1 (5)	H28A—C28—H28B	109.5
C5—C6—Ru1	69.5 (3)	O3—C28—H28C	109.5
C1—C6—Ru1	72.1 (3)	H28A—C28—H28C	109.5
C5—C6—H6	120.9	H28B—C28—H28C	109.5
C1—C6—H6	120.9		
